# Supplementary material for: Models for estimating and projecting global, regional and national prevalence and disease burden of asthma: protocol for a systematic review
Source: BMJ Open. 2017 May 17;7(5):e015441. doi: 10.1136/bmjopen-2016-015441 (PMC5791547; doi:10.1136/bmjopen-2016-015441)
Supplement: Supplementary data [file bmjopen-2016-015441supp001.pdf]

Supplementary APPENDIX

Search Strategies Developed for MEDLINE

1. exp Asthma/
2. Respiratory Sounds/
3. wheez$.mp.
4. Bronchial Spasm/
5. bronchospas$.mp.
6. (bronch$ adj3 spasm$).mp.
7. bronchoconstrict$.mp.
8. asthma$.mp.
9. (antiasthma$ or anti-asthma$).mp.
10. exp Bronchoconstriction/
11. (bronch$ adj3 constrict$).mp.
12. Bronchial Hyperreactivity/
13. Respiratory Hypersensitivity/
14. ((bronchial$ or respiratory or airway$ or lung$) adj3 (hypersensitiv$ or hyperreactiv$ or allerg$ or insufficiency)).mp.
15. or/1-14
16. Epidemiology/
17. morbidity/ or incidence/ or prevalence/ or mortality/
18. (disability-adjusted life years or disability adjusted life years or DALY$).mp.
19. Quality-Adjusted Life Years/ or (quality adjusted life years or QALY$).mp.
20. years lived with disability.mp.
21. years life lost.mp.
22. potential years of life lost.mp.

23. healthy years of life lost.mp.
24. active life expectancy.mp.
25. (disability-free life expectancy or disability free life expectancy).mp.
26. (disability-adjusted life expectancy or disability adjusted life expectancy).mp.
27. (Healthy life expectancy or HALE).mp.
28. (quality adjusted life expectancy or quality-adjusted life expectancy).mp.
29. vital statistics/ or life expectancy/ or life tables/
30. "costs and cost analysis"/ or "cost of illness"/ or health care costs/ or drug costs/ or hospital costs/ or health expenditures/
31. Hospitalization/ or hospitalisation.mp.
32. Primary Health Care/ or Ambulatory Care/ or Emergency Service, Hospital/ or absentee$.mp.
33. burden$.mp.
34. or/16-33
35. model$.mp. or exp models, statistical/ or exp regression analysis/ or odds ratio/ or monte carlo method/ or markov chains/ or Markov.mp.
36. (estimat$ or projection$ or projecting).ti.
37. exp Forecasting/
38. or/35-37
39. 15 and 34 and 38

Search Strategies Developed for EMBASE

1. exp asthma/
2. asthma$.mp.
3. (antiasthma$ or anti-asthma$).mp.
4. abnormal respiratory sound/
5. wheez$.mp.
6. bronchospasm/
7. bronchospas$.mp.
8. (bronch$ adj3 spasm$).mp.
9. bronchoconstrict$.mp.
10. bronchoconstriction/
11. (bronch$ adj3 constrict$).mp.
12. bronchus hyperreactivity/
13. respiratory tract allergy/
14. ((bronchial$ or respiratory or airway$ or lung$) adj3 (hypersensitiv$ or hyperreactiv$ or allerg$ or insufficiency)).mp.
15. or/1-14
16. epidemiology/
17. Prevalence/ or Incidence/ or Morbidity/ or Mortality/
18. burden$.mp.
19. (disability-adjusted life years or disability adjusted life years or DALY$).mp.
20. quality adjusted life year/ or (quality-djusted life years or QALY$).mp.
21. years lived with disability.mp.
22. years life lost.mp.
23. potential years of life lost.mp.

24. healthy years of life lost.mp.
25. Vital statistics/ or Life expectancy/ or Life tables/
26. active life expectancy.mp.
27. (disability-free life expectancy or disability free life expectancy).mp.
28. (disability-adjusted life expectancy or disability adjusted life expectancy).mp.
29. (Healthy life expectancy or HALE).mp.
30. (quality adjusted life expectancy or quality-adjusted life expectancy).mp.
31. "health care cost"/ or "drug cost"/ or "hospital cost"/ or "hospitalization cost"/ or "cost"/ or "cost of illness"/
32. hospitalization/ or Hospitalisation.mp.
33. primary health care/ or medical care/ or emergency care/ or emergency treatment/ or emergency health service/ or ambulatory care/ or absenteeism/
34. or/16-33
35. model/ or loglinear model/ or population model/ or mathematical model/ or proportional hazards model/ or statistical model/ or stochastic model/ or markov chain/ or Markov.mp.
36. (estimat$ or projection$ or projecting).mp.
37. "prediction and forecasting"/
38. or/35-37
39. 15 and 34 and 38

Model evaluation checklist and scoring framework

Checklist

Checklist item	Score obtained (Yes = 1, No = 0)
**Strength**	
**Objective/purpose:**<br>1. Whether there is clear statement about the questions that the model aimed to answer	
**Model formulation and transparency:**<br>Whether there is clear description about<br>1. model structure or model statement (including formula)<br>2. model specification<br>3. model assumptions<br>4. model derivation<br>5. variable used in the model (response, predictors, potential confounders, lag time)<br>6. method of parameter estimation and inference<br>7. model building (variable selection) process<br>8. model diagnostic and adequacy checking<br>9. theoretical ground of the model	
**Data:**<br>Whether there is clear description about<br>1. source of data<br>2. method of data collection (study design)<br>3. sample size and determination process<br>4. method of assessment (data measurement)	
**Model findings:**<br>Whether following information are available<br>1. necessary estimates (prevalence/burden measure/incidence)<br>2. standard errors and 95% confidence intervals of the estimates<br>3. model fit information<br>4. compliance of the model findings with real scenario	
**Model validation:**<br>1. Whether model validation was performed	
**Limitation**	
**Sensitivity analysis:**<br>1. Whether sensitivity analyses was carried out	
**Addressing missing data**<br>1. Whether there is explanation about how missing data were addressed	
**Dissemination and expert involvement:**<br>1. Whether model was disseminated prior to final development of model<br>2. Whether expert opinion were incorporated to develop the model	
**Policymakers involvement:**<br>1. Whether policymakers were involved in the model development<br>2. Whether any policy was recommended on the basis of model derived findings	
**Limitation discussed in the paper:**<br>Whether potential methodological limitations of the model were discussed	

Reproducibility	
**Reproducibility:**<br>1. Whether model is available and accessible to user<br>2. Whether data are available<br>3. Whether codes are available<br>4. Whether user manual to develop the model is available	

Scoring framework

Criteria	Item	Score assigned	Score obtained
Strength	Objectives of the model	1	
	Model formulation and transparency	9	
	Data	4	
	Model findings	4	
	Model validation	1	
Limitation	Sensitivity analysis	1	
	Addressing missing data	1	
	Dissemination and expert involvement	2	
	Policymakers involvement	2	
	Limitation discussed in the paper	1	
Reproducibility	Reproducibility of the model	4	
Total		30	
