# Supplementary material for: Models for estimating and projecting global, regional and national prevalence and disease burden of asthma: protocol for a systematic review
Source: BMJ Open. 2017 May 17;7(5):e015441. doi: 10.1136/bmjopen-2016-015441 (PMC5791547; doi:10.1136/bmjopen-2016-015441)
Supplement: Author's manuscript [file bmjopen-2016-015441.draft_revisions.pdf]

BMJ Open

Models for estimating and projecting global, regional and national prevalence and disease burden of asthma: a scoping review protocol

Journal:	*BMJ Open*
Manuscript ID	bmjopen-2016-015441
Article Type:	Protocol
Date Submitted by the Author:	04-Dec-2016
Complete List of Authors:	Bhuia, Mohammad Romel; Asthma UK Centre for Applied Research, Centre for Medical Informatics, Usher Institute of Population Health Sciences and Informatics, The University of Edinburgh<br>Nwaru, Bright; Asthma UK Centre for Applied Research, Centre for Medical Informatics, Usher Institute of Population Health Sciences and Informatics, The University of Edinburgh; School of Health Sciences, University of Tampere<br>Weir, Christopher; Edinburgh Clinical Trials Unit, Usher Institute of Population Health Sciences and Informatics, The University of Edinburgh<br>Sheikh, Aziz; Asthma UK Centre for Applied Research, Centre for Medical Informatics, Usher Institute of Population Health Sciences and Informatics, The University of Edinburgh
**Primary Subject Heading**:	Epidemiology
Secondary Subject Heading:	Respiratory medicine, Public health, Global health
Keywords:	Asthma < THORACIC MEDICINE, Chronic airways disease < THORACIC MEDICINE, Epidemiology < THORACIC MEDICINE, RESPIRATORY MEDICINE (see Thoracic Medicine), Immunology < THORACIC MEDICINE

SCHOLARONE™
Manuscripts

For peer review only

Models for estimating and projecting global, regional and national prevalence and disease burden of asthma: a scoping review protocol

Mohammad Romel Bhuia[1], Bright I Nwaru[1,2], Christopher J Weir[3], Aziz Sheikh[1]

[1]Asthma UK Centre for Applied Research, Centre for Medical Informatics, Usher Institute of Population Health Sciences and Informatics, The University of Edinburgh, Edinburgh, UK

[2]School of Health Sciences, University of Tampere, Tampere, Finland

[3]Edinburgh Clinical Trials Unit, Usher Institute of Population Health Sciences and Informatics, The University of Edinburgh, Edinburgh, UK

Corresponding author details

Mohammad Romel Bhuia,

Asthma UK Centre for Applied Research

Centre for Medical Informatics

Usher Institute of Population Health Sciences and Informatics

The University of Edinburgh

Room no. 815, Doorway 3, Old Medical School

Teviot Place, Edinburgh EH8 9AG

United Kingdom

E-mail: Mohammad-Romel.Bhuia@ed.ac.uk

Mobile: +44(0)7448680132

Keywords: Asthma, model, prevalence, burden, scoping review

Word count: 1,614

For peer review only

For peer review only - http://bmjopen.bmj.com/site/about/guidelines.xhtml

ABSTRACT

Introduction: Models that have so far been used to estimate and project the prevalence and disease burden of asthma are in most cases inadequately described. We aim systematically to describe and critique the existing models in relation to their strengths, limitations and reproducibility; and determine the appropriate models for estimating and projecting the prevalence and burden of asthma.

Methods: We will identify both published and unpublished (grey literature) primary studies as well as reviews from 1980 to 2016 by searching the following electronic databases: MEDLINE, EMBASE, World Health Organization (WHO) Library and Information Services (WHOLIS – library catalogue of books and reports) and Web of Science Core Collection. We will identify additional studies by searching the reference list of all the retrieved papers and contacting experts. We will include cross-sectional studies that used models for estimating and/or projecting prevalence and disease burden of asthma regarding human population of any age and either sex. Two independent reviewers will assess the studies for inclusion. Key findings from the included studies will be tabulated and a narrative synthesis of the data will be undertaken to scope the relevant evidence base.

Ethics and dissemination: We will not collect any primary data for this review, and hence there is no need for formal National Health Services Research Ethics Committee approval. We will present our findings at scientific conferences and publish the findings in the peer-reviewed scientific journal.

For peer review only

Strengths and limitations of this study

- To the best of our knowledge, this scoping review will be the first study which synthesizes and critiques the existing models for estimating and/or projecting prevalence and burden of asthma to scope the relevant evidence base.
- There is no geographical and language limitations.
- Since this study is a scoping review, a critical narrative synthesis of the models will be undertaken and no formal quality assessment and risk of bias assessment of the included studies will be performed.

For peer review only

For peer review only - http://bmjopen.bmj.com/site/about/guidelines.xhtml

BACKGROUND

Asthma is now one of the commonest long-term conditions in the world and it is responsible for substantial morbidity and in some cases mortality.[1] The overall worldwide trend in the prevalence of asthma appears to have plateaued in some parts of the world; while it is still increasing in some countries.[2] Asthma has been ranked as the 14th most important cause of years lived with disability (YLDs) in the world[3] and it accounts for 1% of all disability-adjusted life years (DALYs) lost globally.[4]

The societal and healthcare costs attributed to asthma are also high across different world regions: for instance, across Europe, the cost of persistent asthma among those aged 15 to 64 years was estimated in 2010 values at about 19.3 billion Euros;[5] in Asia-Pacific region, the total annual per-patient societal costs of asthma varied from 184 US dollars in Vietnam to 1,189 US dollars in Hong Kong (2000 rates).[6] Likewise, at national levels, asthma imposes considerable economic burden to the health care system besides its negative impact on the quality of life of individuals and families.[7-8] For example, recent estimates found that asthma costs at least 1.1 billion pounds sterling per year to United Kingdom and its member nations.[9] Whilst, in the United States, the total cost attributed to asthma in 2007 was estimated about 56 billion US dollars.[10]

Although varying estimates of asthma prevalence and burden at the national, regional, and global levels have been reported in the published literature,[3, 11-16] almost all appear to have major limitations in terms of inadequacy of the analytical approach used and lack of reproducibility.[17-19] There is therefore a need for generating valid and reproducible estimates of disease prevalence and burden of asthma to inform evidence-based policy deliberations. Developing transparent processes for generating the national, regional, and

For peer review only - http://bmjopen.bmj.com/site/about/guidelines.xhtml

global estimates of prevalence and burden of asthma will thus enhance reproducibility across settings and will allow reliable projection of future estimates.

In health care policy, a model can be defined as a logical mathematical framework or analytical methodology that integrates theories and data to draw inferences regarding parameters of interest to clinicians and decision makers.[20-21] Existing models for estimating and projecting prevalence and disease burden of asthma are in most cases poorly described thereby limiting the opportunity to assess their reproducibility. In order to gain a better appreciation of the performance of existing models and their capacity for reproducibility in estimating the burden of asthma, a systematic appraisal of the underlying evidence base is required.[22] The aims of this scoping review are therefore to: i) systematically describe and critique the existing models for estimating and/or projecting the global, regional and national prevalence and burden of asthma in relation to their strengths, limitations and reproducibility, and ii) determine the appropriate models for estimating and projecting the prevalence and burden of asthma.

METHODS

Arksey and O'Malley[23] proposed a framework for conducting scoping reviews, which was later amended by Levac et al.[24] to offer more explicit detail about what should be done at each stage of the review process for ensuring transparency and accuracy of the review process. Both of these frameworks have been considered in the development of this protocol. Development of each part of this protocol has also been guided by 'The Joanna Briggs Institute Reviewers' Manual 2015 for scoping reviews'.[25]

For peer review only - http://bmjopen.bmj.com/site/about/guidelines.xhtml

Identifying relevant studies

A comprehensive literature search will be undertaken to identify both published and unpublished (grey literature) primary studies as well as reviews. The preliminary search strategy has been developed for MEDLINE (see supplementary Appendix) in consultation with a senior medical librarian at The University of Edinburgh and this will be adapted in searching other databases.

Search strategy

Following the recommendation of 'The Joanna Briggs Institute Reviewers' Manual 2015 for scoping reviews',[25] the search for this review will be conducted in three steps. First, a primary limited search will be carried out in two databases namely MEDLINE and EMBASE. This primary search will be then followed by an analysis of the text words contained in the title and abstract of retrieved papers, and of the index terms used to describe the articles. Using all identified keywords and index terms, a second search will be undertaken to the following electronic databases: MEDLINE, EMBASE, World Health Organization (WHO) Library and Information Services (WHOLIS – library catalogue of books and reports) and Web of Science Core Collection. Finally, the reference list of all the retrieved papers will be searched for additional studies. Over and above this, we will contact a panel of experts in an attempt to identify additional unpublished or in progress studies.

Inclusion criteria

According to the recommendation of the 'The Joanna Briggs Institute Reviewers' Manual 2015 for scoping reviews',[25] for ensuring the scope of the review remains broad, a PCC (i.e. Population, Concept and Context) mnemonic is used to categorise the inclusion criteria for this review. This is in keeping with the aims of scoping reviews less restrictive than the

For peer review only - http://bmjopen.bmj.com/site/about/guidelines.xhtml

PICO (i.e. Population, Intervention, Control and Outcomes) framework that is traditionally used in systematic reviews. This is detailed below.

Population

Human populations of any age and either sex will constitute the population of interest in this review.

Concept

Any study that developed models for estimating and/or projecting prevalence and disease burden of asthma will be included in this review. Studies that estimated prevalence and disease burden without modelling will be excluded from this review. Models that estimate individual risk rather than population benefits, such as decision analytic models, individual prognostic models will be excluded from the review. Moreover, studies with models that simply describe animals, clinical series and cell lines will be excluded. Comparative intervention studies will also not be included in the review. We will include the studies from January 1980 to October 2016. The start date has been set up from the time when modelling techniques started to be applied broadly to study the epidemic of non-communicable diseases.[26]

Context

Research articles from any country and any setting will be included in this review. Potential sources of evidence such as original research articles and review articles including systematic reviews, meta-analyses and meta-syntheses of cross-sectional studies will be included.

For peer review only - http://bmjopen.bmj.com/site/about/guidelines.xhtml

Study selection

Two reviewers will independently check and screen the titles and abstracts of identified articles against the inclusion criteria. Full-text copies of potentially relevant studies will be obtained and assessed by two independent reviewers on the basis of their eligibility for inclusion. Any discrepancies will be resolved by discussion, and disagreements will be arbitrated by a third reviewer.

Data extraction

Keeping consistency with the objectives of this scoping review, tabular summary of information and qualitative thematic analysis will be incorporated. A draft data extraction form has been developed to keep record of the key information of the sources. During the review process, this draft will be refined and the data extraction form will be updated accordingly. The data extraction form will be pre-piloted.

Collating, summarising and reporting the results

Key findings from the included studies will be tabulated. Papers will be grouped according to the type of models that they reported. The tabular presentation of the key findings will include distribution of studies by name of the authors, year of publication, study population/data, model type used, aims, methodology adopted, key findings (evidence established) of the study and identified research gaps. A clear description will be provided for each of the reported categories. Moreover, a narrative synthesis of the data will be undertaken to summarise the overall evidence.

For peer review only - http://bmjopen.bmj.com/site/about/guidelines.xhtml

CONCLUSIONS

To the best of our knowledge no review has been undertaken yet to appraise the models for estimating and projecting the global, regional and national prevalence and disease burden of asthma; this scoping review will be the first study which synthesises the existing models for estimating and projecting prevalence and burden of asthma, and map the appropriate models that will subsequently be used to obtain current estimates and project future trend of global, regional and national prevalence and disease burden of asthma.

ETHICS AND DISSEMINATION

We will not collect any primary data for this review, and hence there is no need for formal National Health Service (NHS) Research Ethics Committee review. This work is however subject to Institutional Review Board oversight by The University of Edinburgh's Centre for Population Health Sciences. Findings from this review will be presented at scientific conferences and be published in the peer-reviewed scientific journal.

Acknowledgement

We thankful to Marshall Dozier, Senior Liaison Librarian for the College of Medicine and Veterinary Medicine, The University of Edinburgh for her support in developing the search strategies. We also grateful to Dr Susannah McLean, Andrew Stoddart and the Asthma UK Centre for Applied Research.

Competing interests

None declared.

For peer review only

Funding

MRB is supported by the College of Medicine & Veterinary Medicine, The University of Edinburgh and the Farr Institute, UK. BN and AS are also supported by the Farr Institute, UK. The Farr Institute is funded by a consortium of funders led by the Medical Research Council (MRC).

Contributors

AS conceived the idea for this work. MRB drafted the protocol under the supervision of AS, BIN and CJW. The draft was revised according to several rounds of critical comments from AS, BIN and CJW. All the authors will be involved in the scoping review process.

For peer review only

For peer review only - http://bmjopen.bmj.com/site/about/guidelines.xhtml

REFERENCES

1. Global Asthma Network. The Global Asthma Report 2014, 2014.

2. Anandan C, Nurmatov U, van Schayck OC, et al. Is the prevalence of asthma declining? Systematic review of epidemiological studies. *Allergy* 2010;**65**(2):152-67.

3. Vos T, Flaxman AD, Naghavi M, et al. Years lived with disability (YLDs) for 1160 sequelae of 289 diseases and injuries 1990-2010: a systematic analysis for the Global Burden of Disease Study 2010. *Lancet* 2012;**380**(9859):2163-96.

4. Masoli M, Fabian D, Holt S, et al. The global burden of asthma: executive summary of the GINA Dissemination Committee report. *Allergy* 2004;**59**(5):469-78.

5. Accordini S, Corsico AG, Braggion M, et al. The cost of persistent asthma in Europe: an international population-based study in adults. *Int Arch Allergy Immunol* 2013;**160**(1):93-101.

6. C. K. W. Lai Y-YK, S-H. Kuo, M. Spencer, A. E. Williams on behalf of the, Committee AIaRiAPS. Cost of asthma in the Asia-Pacific region. *Eur Respir Rev* 2006;**15**(98):10-16.

7. Stevens CA, Turner D, Kuehni CE, et al. The economic impact of preschool asthma and wheeze. *Eur Respir J* 2003;**21**(6):1000-6.

8. Bahadori K, Doyle-Waters MM, Marra C, et al. Economic burden of asthma: a systematic review. *BMC Pulm Med* 2009;**9**:24.

9. Mukherjee M, Stoddart A, Gupta RP, et al. The epidemiology, healthcare and societal burden and costs of asthma in the UK and its member nations: analyses of standalone and linked national databases. *BMC Med* 2016;**14**(1):113.

For peer review only

For peer review only - http://bmjopen.bmj.com/site/about/guidelines.xhtml

10. Barnett SB, Nurmagambetov TA. Costs of asthma in the United States: 2002-2007. *J Allergy Clin Immunol* 2011;**127**(1):145-52.

11. To T, Stanojevic S, Moores G, et al. Global asthma prevalence in adults: findings from the cross-sectional world health survey. BMC Public Health 2012;**12**:204.

12. Sembajwe G, Cifuentes M, Tak SW, et al. National income, self-reported wheezing and asthma diagnosis from the World Health Survey. *Eur Respir J* 2010;**35**(2):279-86.

13. Lai CK, Beasley R, Crane J, et al. Global variation in the prevalence and severity of asthma symptoms: phase three of the International Study of Asthma and Allergies in Childhood (ISAAC). Thorax 2009;**64**(6):476-83.

14. Basagana X, Sunyer J, Kogevinas M, et al. Socioeconomic status and asthma prevalence in young adults: the European Community Respiratory Health Survey. *Am J Epidemiol* 2004;**160**(2):178-88.

15. Worldwide variations in the prevalence of asthma symptoms: the International Study of Asthma and Allergies in Childhood (ISAAC). *Eur Respir J* 1998;**12**(2):315-35.

16. Variations in the prevalence of respiratory symptoms, self-reported asthma attacks, and use of asthma medication in the European Community Respiratory Health Survey (ECRHS). *Eur Respir J* 1996;**9**(4):687-95.

17. Adeloye D, Chan KY, Rudan I, et al. An estimate of asthma prevalence in Africa: a systematic analysis. *Croat Med J* 2013;**54**(6):519-31.

18. Galobardes B, Sunyer J, Anto JM, et al. Effect of the method of administration, mail or telephone, on the validity and reliability of a respiratory health questionnaire. The Spanish Centers of the European Asthma Study. *J Clin Epidemiol* 1998;**51**(10):875-81.

For peer review only

For peer review only - http://bmjopen.bmj.com/site/about/guidelines.xhtml

19. Anand S, Hanson K. Disability-adjusted life years: a critical review. *J Health Econ* 1997;**16**(6):685-702.

20. Unal B, Capewell S, Critchley JA. Coronary heart disease policy models: a systematic review. BMC Public Health 2006;**6**:213.

21. Weinstein MC, O'Brien B, Hornberger J, et al. Principles of good practice for decision analytic modeling in health-care evaluation: report of the ISPOR Task Force on Good Research Practices--Modeling Studies. *Value Health* 2003;**6**(1):9-17.

22. Peng R. The reproducibility crisis in science: A statistical counterattack. *Significance* 2015;**12**(3):30-32.

23. Arksey H, O'Malley L. Scoping studies: towards a methodological framework. *Int J Soc Res Methodol* 2005;**8**(1):19-32.

24. Levac D, Colquhoun H, O'Brien KK. Scoping studies: advancing the methodology. *Implement Sci* 2010;**5**(1):1.

25. Peters M, Godfrey C, McInerney P, et al. *The Joanna Briggs Institute Reviewers' Manual 2015: Methodology for JBI Scoping Reviews*: The Joanna Briggs Institute, 2015.

26. Gunning-Schepers L. The health benefits of prevention: a simulation approach. *Health Policy* 1989;**12**(1-2):1-255.

For peer review only

For peer review only - http://bmjopen.bmj.com/site/about/guidelines.xhtml

APPENDIX: Search Strategies Developed for MEDLINE

1. exp Asthma/
2. asthma$.mp
3. (antiasthma$ or anti-asthma$).mp
4. Respiratory Sounds/
5. wheez$.mp
6. Bronchial Spasm/
7. bronchospas$.mp.
8. (bronch$ adj3 spasm$).mp
9. bronchoconstrict$.mp
10. exp Bronchoconstriction/
11. (bronch$ adj3 constrict$).mp
12. Bronchial Hyperreactivity/
13. Respiratory Hypersensitivity/
14. ((bronchial$ or respiratory or airway$ or lung$) adj3 (hypersensitiv$ or hyperreactiv$ or allerg$ or insufficiency)).mp
15. or/1-14
16. Epidemiology/
17. Morbidity/ or Incidence/ or Prevalence/
18. burden$.mp.
19. Global Health/ or gbd.mp.
20. (disability-adjusted life years or disability adjusted life years or DALY$).mp.
21. Quality-Adjusted Life Years/ or (quality adjusted life years or QALY$).mp.
22. years lived with disability.mp.
23. years life lost.mp.

For peer review only

For peer review only - http://bmjopen.bmj.com/site/about/guidelines.xhtml

24. potential years of life lost.mp.

25. healthy years of life lost.mp.

26. Vital statistics/ or Life expectancy/ or Life tables/

27. active life expectancy.mp.

28. disability-free life expectancy.mp.

29. disability-adjusted life expectancy.mp.

30. HALE.mp.

31. quality adjusted life expectancy.mp.

32. "Quality of Life"/

33. exp Mortality/

34. exp "Costs and Cost Analysis"/

35. Health Care Rationing/

36. Hospitalization/ or hospitalisation.mp.

37. House calls/ or office visits/ or "referral and consultation"/

38. or/16-37

39. (Estimate$ or estimation$ or estimating or estimat*).mp.

40. exp Forecasting/

41. (projection$ or project* or projecting).ti,ab.

42. (prediction$ or predicting or predict*).mp.

43. (model* or modelling or modeling).mp.

44. exp Models, statistical/ or models, economic/ or models, econometric/ or monte carlo method/ or exp regression analysis/ or markov chains/ or odds ratio/ or Markov.mp.

45. or/39-44

46. 15 and 38 and 45

For peer review only - http://bmjopen.bmj.com/site/about/guidelines.xhtml

BMJ Open

Models for estimating and projecting global, regional and national prevalence and disease burden of asthma: protocol for a systematic review

Journal:	*BMJ Open*
Manuscript ID	bmjopen-2016-015441.R1
Article Type:	Protocol
Date Submitted by the Author:	31-Jan-2017
Complete List of Authors:	Bhuia, Mohammad Romel; Asthma UK Centre for Applied Research, Centre for Medical Informatics, Usher Institute of Population Health Sciences and Informatics, The University of Edinburgh<br>Nwaru, Bright; Asthma UK Centre for Applied Research, Centre for Medical Informatics, Usher Institute of Population Health Sciences and Informatics, The University of Edinburgh; School of Health Sciences, University of Tampere<br>Weir, Christopher; Edinburgh Clinical Trials Unit, Usher Institute of Population Health Sciences and Informatics, The University of Edinburgh<br>Sheikh, Aziz; Asthma UK Centre for Applied Research, Centre for Medical Informatics, Usher Institute of Population Health Sciences and Informatics, The University of Edinburgh
**Primary Subject Heading**:	Epidemiology
Secondary Subject Heading:	Respiratory medicine, Public health, Global health
Keywords:	Asthma < THORACIC MEDICINE, Epidemiology < THORACIC MEDICINE, Model, Prevalence, Burden, Systematic review

SCHOLARONE™
Manuscripts

For peer review only - http://bmjopen.bmj.com/site/about/guidelines.xhtml

Models for estimating and projecting global, regional and national prevalence and disease burden of asthma: protocol for a systematic review

Mohammad Romel Bhuia[1], Bright I Nwaru[1,2], Christopher J Weir[3], Aziz Sheikh[1]

[1]Asthma UK Centre for Applied Research, Centre for Medical Informatics, Usher Institute of Population Health Sciences and Informatics, The University of Edinburgh, Edinburgh, UK

[2]School of Health Sciences, University of Tampere, Tampere, Finland

[3]Edinburgh Clinical Trials Unit, Usher Institute of Population Health Sciences and Informatics, The University of Edinburgh, Edinburgh, UK

Corresponding author details

Mohammad Romel Bhuia

Asthma UK Centre for Applied Research

Centre for Medical Informatics

Usher Institute of Population Health Sciences and Informatics

The University of Edinburgh

Room no. 815, Doorway 3, Old Medical School

Teviot Place, Edinburgh EH8 9AG

United Kingdom

E-mail: Mohammad-Romel.Bhuia@ed.ac.uk

Tel: +44(0)131 650 3178

Keywords: Asthma, model, prevalence, burden, systematic review

Word count: 1,880

For peer review only - http://bmjopen.bmj.com/site/about/guidelines.xhtml

ABSTRACT

Introduction: Models that have so far been used to estimate and project the prevalence and disease burden of asthma are in most cases inadequately described and irreproducible. We aim systematically to describe and critique the existing models in relation to their strengths, limitations and reproducibility; and determine the appropriate models for estimating and projecting the prevalence and burden of asthma.

Methods: We will search the following electronic databases to identify relevant literature published from 1980 to 2017: MEDLINE, EMBASE, World Health Organization Library, and Web of Science Core Collection. We will identify additional studies by searching the reference list of all the retrieved papers and contacting experts. We will include observational studies that used models for estimating and/or projecting prevalence and disease burden of asthma regarding human population of any age and sex. Two independent reviewers will assess the studies for inclusion and extract data from included papers. Data items will include: authors' names, publication year, study aims, data source and time period, study population, asthma outcomes, study methodology, model type, model settings, study variables, methods of model derivation, methods of parameter estimation and/or projection, model fit information, key findings, and identified research gaps. A detailed critical narrative synthesis of the models will be undertaken in relation to their strengths, limitations and reproducibility. A quality assessment checklist and scoring framework will be used to determine the appropriate models for estimating and projecting the prevalence and burden of asthma.

Ethics and dissemination: We will not collect any primary data for this review, and hence there is no need for formal National Health Services Research Ethics Committee

For peer review only

approval. We will present our findings at scientific conferences and publish the findings in the peer-reviewed scientific journal.

Strengths and limitations of this study

- To the best of our knowledge, this is the first systematic review to synthesise and critique existing models for estimating and/or projecting the prevalence and burden of asthma to scope the relevant evidence base.
- There is no geographical and language limitations.
- Comprehensive and highly sensitive search strategies, identification of studies form leading medical and public health databases, and involvement a panel of expert will ensure quality of underlying evidence base.
- Panel of experts should be consulted due to lack of standard reporting guidelines for modelling studies.

For peer review only

BACKGROUND

Asthma is now one of the commonest long-term conditions in the world and it is responsible for substantial morbidity and in some cases mortality.[1] The overall worldwide trend in the prevalence of asthma appears to have plateaued in some parts of the world; while it is still increasing in some countries.[2] Asthma has been ranked as the 14th most important cause of years lived with disability (YLDs) in the world[3] and it accounts for 1% of all disability-adjusted life years (DALYs) lost globally.[4]

The societal and healthcare costs attributed to asthma are also high across different world regions: for instance, across Europe, the cost of persistent asthma among those aged 15 to 64 years was estimated in 2010 values at about 19.3 billion Euros;[5] in Asia-Pacific region, the total annual per-patient societal costs of asthma varied from 184 US dollars in Vietnam to 1,189 US dollars in Hong Kong (2000 rates).[6] Likewise, at national levels, asthma imposes considerable economic burden to the health care system besides its negative impact on the quality of life of individuals and families.[7-8] For example, recent estimates found that asthma costs at least 1.1 billion pounds sterling per year to United Kingdom and its member nations.[9] Whilst, in the United States, the total cost attributed to asthma in 2007 was estimated about 56 billion US dollars.[10]

Although varying estimates of asthma prevalence and burden at the national, regional, and global levels have been reported in the published literature,[3, 11-16] almost all appear to have major limitations in terms of inadequacy of the analytical approach used and lack of reproducibility.[17-19] There is therefore a need for generating valid and reproducible estimates of disease prevalence and burden of asthma to inform evidence-based policy deliberations. Developing transparent processes for generating the national, regional, and

For peer review only - http://bmjopen.bmj.com/site/about/guidelines.xhtml

global estimates of prevalence and burden of asthma will thus enhance reproducibility across settings and will allow reliable projection of future estimates.

In health care policy, a model can be defined as a logical mathematical framework or analytical methodology that integrates theories and data to draw inferences regarding parameters of interest to clinicians and decision makers.[20-21] Models are widely used to estimate disease burden[9, 22-24], trend in prevalence[25-26] and future projections[27] of different epidemiological characteristics of asthma . Although current prevalence can be estimated without applying a model, many studies[15, 17, 28-31] estimated asthma prevalence applying modelling techniques, particularly with the aim of adjusting for certain vital population characteristics, such as age, sex, time, geography, and other contextual parameters that may vary across studies. For example, the International Study of Asthma and Allergies in Childhood (ISAAC) Steering Committee applied generalised linear mixed model to estimate the global prevalence of asthma in order to adjust for within-country and between-country variations.[15] Adeloye et al.[17] applied a non-linear model to estimate regional (Africa) prevalence of asthma. The Global Burden of Disease (GBD) studies also developed some models (DisMod, DisMod II, DisMod-MR, DisMod-MR 2.1) for estimating disease prevalence.[28, 32] However, existing models for estimating and projecting prevalence and disease burden of asthma are in most cases poorly described thereby limiting the opportunity to assess their reproducibility. In order to gain a better appreciation of the performance of existing models and their capacity for reproducibility in estimating the burden of asthma, a systematic appraisal of the underlying evidence base is required.[33]

For peer review only - http://bmjopen.bmj.com/site/about/guidelines.xhtml

Objectives

The aims of this systematic review are to: i) systematically describe and critique the existing models for estimating and/or projecting the global, regional and national prevalence and burden of asthma in relation to their strengths, limitations and reproducibility, and ii) determine the appropriate models for estimating and projecting the prevalence and burden of asthma.

METHODS

Eligibility criteria

Types of studies

Any study that developed models for estimating and/or projecting prevalence and disease burden of asthma will be included in this review. Studies that estimated prevalence and disease burden without modelling will be excluded. Models that estimated individual risk rather than population benefits, such as decision analytic models, individual prognostic models will be excluded. Moreover, studies with models that simply describe animals, clinical series and cell lines will be excluded. Comparative intervention studies will also be excluded. Potential sources of evidence such as original research articles and review articles including systematic reviews, meta-analyses and meta-syntheses of observational studies will be included.

Participants

Eligible participants in this review will include human populations of any age and either sex.

For peer review only - http://bmjopen.bmj.com/site/about/guidelines.xhtml

For peer review only

Years considered

We will include studies from January 1980 to February 2017. The start date has been set up from the time when modelling techniques started to be applied broadly to study the epidemic of non-communicable diseases.[34]

Setting

Research articles from any country and any setting (urban/rural) will be included in this review.

Language

There will be no language restrictions and, where possible, we will translate the literature published in languages other than English.

Information sources

Database searches and other sources to identify studies

We will conduct searches to identify both published and unpublished modelling studies in the following electronic databases: MEDLINE, EMBASE, World Health Organization (WHO) Library and Information Services (WHOLIS – library catalogue of books and reports) and Web of Science Core Collection. The reference lists of all the included papers will be searched for additional studies. We will also contact a panel of experts in an attempt to identify additional unpublished or in progress studies.

For peer review only

For peer review only - http://bmjopen.bmj.com/site/about/guidelines.xhtml

Search strategy

A comprehensive literature search will be undertaken to identify both published and unpublished (grey literature) primary studies as well as reviews. The search strategy has been developed for searching literature in MEDLINE and EMBASE (see supplementary Appendix) in consultation with a senior medical librarian at The University of Edinburgh and this will be adapted in searching other databases. The search terms include the concepts of 'modelling', 'prevalence and disease burden' and 'asthma'.

Study records

Data management

The retrieved records from all databases will be exported to Endnote Library, which will be used throughout the review for study screening, deduplication and overall management of the retrieved records.

Selection process

Two reviewers will independently check and screen the titles and abstracts of identified articles against the inclusion criteria. Full-text copies of potentially relevant studies will be obtained and assessed by two independent reviewers on the basis of their eligibility for inclusion. Any discrepancies will be resolved by discussion, and disagreements will be arbitrated by a third reviewer.

Data extraction

A data extraction form will be used to extract relevant data from included studies. We have developed a draft data extraction form. During the review process, this draft will be refined

For peer review only - http://bmjopen.bmj.com/site/about/guidelines.xhtml

and the data extraction form will be updated accordingly. The data extraction form will be pre-piloted prior to full use in the review. Data extraction will be performed independently by two reviewers.

Data items

Information regarding different components of the model will be recorded to get a comprehensive picture of the model. The following data items will be extracted from each study: authors' names; publication year; study aims; data source and time period; study population; asthma outcomes (prevalence/disease burden); study methodology; model type; model settings; model formulation (structure, specification, assumptions, methods of model derivation, methods of parameter estimation and/or projection, theoretical basis of the models) study variables; availability of data and codes; findings from the models; model fit information; key findings of the study; and identified research gaps. Information regarding the model availability, transparency, sensitivity analysis, model validation, addressing missing data, policymakers involvement, dissemination and expert involvement, limitation discussed, and reproducibility of the model will also be extracted. Descriptive tables will be used to tabulate these items and to summarise the literature. The systematic review will be reported following the guidelines of the PRISMA checklist.[35]

Outcomes and prioritisation

The outcomes which are of interest include prevalence and disease burden of asthma. There are various measures available to quantify disease burden. All the established measures of disease burden will be considered in this review. Primary and secondary outcomes are categorised as follows.

For peer review only

Primary outcomes

1. prevalence of asthma;

2. different measures of disease burden of asthma. The measures are:

— disability-adjusted life years (DALYs); quality-adjusted life years (QALYs); years lived with disability (YLDs); mortality; health care cost (cost of illness, drug cost, hospital cost/hospitalisation cost); life expectancy; primary care; ambulatory care; emergency visit; absentees; years life lost; potential years of life lost; healthy years of life lost; active life expectancy; disability-free life expectancy; disability-adjusted life expectancy; Healthy life expectancy (HALE); quality adjusted life expectancy etc.

Secondary outcomes

1. incidence of asthma

Risk of bias in individual studies

To the best of our knowledge, there is no existing quality appraisal tool to assess quality of models. So, we have drawn on first principles and adapted relevant sections from pertinent reporting guidelines[36] and other guidelines for good practice in modelling studies[20-21, 37-38] to develop our own model evaluation framework. This will involve independent assessment of the strengths and limitations of the models on the basis of model structure, specification, assumptions, sensitivity analysis, model validation, dealing with missing data, theoretical basis of the models, incorporation of confounding factors and lag times, and whether potential methodological limitations are described. Reproducibility of the model will be assessed on the basis of availability of the models, data, codes and methods of parameter estimation. A model will be categorised as reproducible if it is possible to obtain same output as reported in the paper after running that model on the provided data.

For peer review only - http://bmjopen.bmj.com/site/about/guidelines.xhtml

To evaluate the models used in included studies and to identify the best model, we have prepared a checklist of items and formulated a scoring strategy (see supplementary Appendix) that we will use for these purposes. Prior to use of the checklist, we plan to consult with a panel of experts in the field of modelling studies to gain their insights and criticisms of the checklist; we will then integrate feedback collated in preparing the final version of the checklist to be used in our study.

Data synthesis

A tabular summary of the data will be presented to summarise overall evidence. A detailed critical narrative synthesis of the models will be undertaken regarding their strengths, limitations and reproducibility.

Protocol registration

A detailed protocol for the systematic review will be registered with the International Prospective Register of Systematic Reviews (PROSPERO) prior to commencing the review according to the Preferred Reporting Items for Systematic review and Meta Analysis Protocols (PRISMA-P) 2015 statement.[39]

CONCLUSIONS

To the best of our knowledge no review has been undertaken yet to appraise the models for estimating and projecting the global, regional and national prevalence and disease burden of asthma. This systematic review is therefore the first study to synthesise existing models for estimating and projecting prevalence and burden of asthma. The review will also map the appropriate models that will subsequently be used to obtain current estimates and project future trend of global, regional and national prevalence and disease burden of asthma.

For peer review only

ETHICS AND DISSEMINATION

We will not collect any primary data for this review, and hence there is no need for formal National Health Service (NHS) Research Ethics Committee review. This work is however subject to Institutional Review Board oversight by The University of Edinburgh's Centre for Population Health Sciences. Findings from the review will be presented at scientific conferences and be published in the peer-reviewed scientific journal.

Acknowledgement

We thankful to Marshall Dozier, Senior Liaison Librarian for the College of Medicine and Veterinary Medicine, The University of Edinburgh for her support in developing the search strategies. We also thankful to Dr Susannah McLean for providing necessary suggestions and resources regarding modelling studies, Dr Niall Anderson for his suggestions in developing scoring framework to appraise the models, Andrew Stoddart for suggesting search terms related to economic burden of asthma and the Asthma UK Centre for Applied Research for providing platform to develop this protocol.

Competing interests

None declared.

Funding

MRB is supported by the College of Medicine & Veterinary Medicine, The University of Edinburgh and the Farr Institute, UK. BN and AS are also supported by the Farr Institute, UK. The Farr Institute is funded by a consortium of funders led by the Medical Research Council (MRC).

Contributors

AS conceived the idea for this work. MRB drafted the protocol under the supervision of AS, BN and CW. The draft was revised according to several rounds of critical comments from AS, BN and CW. All the authors will be involved in the systematic review process.

For peer review only - http://bmjopen.bmj.com/site/about/guidelines.xhtml

REFERENCES

1. Global Asthma Network. The Global Asthma Report 2014, 2014.

2. Anandan C, Nurmatov U, van Schayck OC, et al. Is the prevalence of asthma declining? Systematic review of epidemiological studies. *Allergy* 2010;**65**(2):152-67.

3. Vos T, Flaxman AD, Naghavi M, et al. Years lived with disability (YLDs) for 1160 sequelae of 289 diseases and injuries 1990-2010: a systematic analysis for the Global Burden of Disease Study 2010. *Lancet* 2012;**380**(9859):2163-96.

4. Masoli M, Fabian D, Holt S, et al. The global burden of asthma: executive summary of the GINA Dissemination Committee report. *Allergy* 2004;**59**(5):469-78.

5. Accordini S, Corsico AG, Braggion M, et al. The cost of persistent asthma in Europe: an international population-based study in adults. *Int Arch Allergy Immunol* 2013;**160**(1):93-101.

6. Lai C, Kim Y, Kuo S, et al. Cost of asthma in the Asia-Pacific region. *Eur Respir Rev* 2006;**15**(98):10-16.

7. Stevens CA, Turner D, Kuehni CE, et al. The economic impact of preschool asthma and wheeze. *Eur Respir J* 2003;**21**(6):1000-6.

8. Bahadori K, Doyle-Waters MM, Marra C, et al. Economic burden of asthma: a systematic review. *BMC Pulm Med* 2009;**9**:24.

9. Mukherjee M, Stoddart A, Gupta RP, et al. The epidemiology, healthcare and societal burden and costs of asthma in the UK and its member nations: analyses of standalone and linked national databases. *BMC Med* 2016;**14**(1):113.

10. Barnett SB, Nurmagambetov TA. Costs of asthma in the United States: 2002-2007. *J Allergy Clin Immunol* 2011;**127**(1):145-52.

For peer review only - http://bmjopen.bmj.com/site/about/guidelines.xhtml

11. To T, Stanojevic S, Moores G, et al. Global asthma prevalence in adults: findings from the cross-sectional world health survey. BMC Public Health 2012;**12**:204.

12. Sembajwe G, Cifuentes M, Tak SW, et al. National income, self-reported wheezing and asthma diagnosis from the World Health Survey. *Eur Respir J* 2010;**35**(2):279-86.

13. Lai CK, Beasley R, Crane J, et al. Global variation in the prevalence and severity of asthma symptoms: phase three of the International Study of Asthma and Allergies in Childhood (ISAAC). Thorax 2009;**64**(6):476-83.

14. Basagana X, Sunyer J, Kogevinas M, et al. Socioeconomic status and asthma prevalence in young adults: the European Community Respiratory Health Survey. *Am J Epidemiol* 2004;**160**(2):178-88.

15. ISAAC Steering Committee. Worldwide variations in the prevalence of asthma symptoms: the International Study of Asthma and Allergies in Childhood (ISAAC). *Eur Respir J* 1998;**12**(2):315-35.

16. European Community Respiratory Health Survey. Variations in the prevalence of respiratory symptoms, self-reported asthma attacks and use of asthma medication in the European Community Respiratory Health Survey (ECRHS). *Eur Respir J* 1996;**9**(4):687-95.

17. Adeloye D, Chan KY, Rudan I, et al. An estimate of asthma prevalence in Africa: a systematic analysis. *Croat Med J* 2013;**54**(6):519-31.

18. Galobardes B, Sunyer J, Anto JM, et al. Effect of the method of administration, mail or telephone, on the validity and reliability of a respiratory health questionnaire. The Spanish Centers of the European Asthma Study. *J Clin Epidemiol* 1998;**51**(10):875-81.

For peer review only

For peer review only - http://bmjopen.bmj.com/site/about/guidelines.xhtml

19. Anand S, Hanson K. Disability-adjusted life years: a critical review. *J Health Econ* 1997;**16**(6):685-702.

20. Unal B, Capewell S, Critchley JA. Coronary heart disease policy models: a systematic review. *BMC Public Health* 2006;**6**:213.

21. Weinstein MC, O'Brien B, Hornberger J, et al. Principles of good practice for decision analytic modeling in health-care evaluation: report of the ISPOR Task Force on Good Research Practices--Modeling Studies. *Value Health* 2003;**6**(1):9-17.

22. Bavbek S, Mungan D, Turktas H, et al. A cost-of-illness study estimating the direct cost per asthma exacerbation in Turkey. *Respir Med* 2011;105(4):541-8.

23. Blasco Bravo AJ, Perez-Yarza EG, Lazaro y de Mercado P, et al. Cost of childhood asthma in Spain: a cost evaluation model based on the prevalence. *An Pediatr (Barc)* 2011;74(3):145-53.

24. Kassebaum NJ, Arora M, Barber RM, et al. Global, regional, and national disability-adjusted life-years (DALYs) for 315 diseases and injuries and healthy life expectancy (HALE), 1990-2015: a systematic analysis for the Global Burden of Disease Study 2015. *Lancet (London, England)* 2016;388(10053):1603-58.

25. Ma YC, Lin CC, Yang SY, et al. Time Trend Analysis of the Prevalence and Incidence of Diagnosed Asthma and Traditional Chinese Medicine Use among Adults in Taiwan from 2000 to 2011: A Population-Based Study. *PloS one* 2015;10(10):e0140318.

26. Ones U, Akcay A, Tamay Z, et al. Rising trend of asthma prevalence among Turkish schoolchildren (ISAAC phases I and III). *Allergy* 2006;61(12):1448-53.

27. To T, Stanojevic S, Feldman R, et al. Is asthma a vanishing disease? A study to forecast the burden of asthma in 2022. *BMC Public Health* 2013;13:254.

For peer review only

For peer review only - http://bmjopen.bmj.com/site/about/guidelines.xhtml

28. Vos T, Allen C, Arora M, et al. Global, regional, and national incidence, prevalence, and years lived with disability for 310 diseases and injuries, 1990-2015: a systematic analysis for the Global Burden of Disease Study 2015. *Lancet (London, England)* 2016;388(10053):1545-602.

29. Tomasallo CD, Hanrahan LP, Tandias A, et al. Estimating Wisconsin asthma prevalence using clinical electronic health records and public health data. *American Journal of Public Health* 2014;104(1):e65-73.

30. Solis Soto MT, Patino A, Nowak D, et al. Prevalence of asthma, rhinitis and eczema symptoms in rural and urban school-aged children from Oropeza Province - Bolivia: a cross-sectional study. *BMC Pulm Med* 2014;14:40.

31. McHugh MK, Symanski E, Pompeii LA, et al. Prevalence of asthma among adult females and males in the United States: results from the National Health and Nutrition Examination Survey (NHANES), 2001-2004. *J Asthma* 2009;46(8):759-66.

32. Barendregt JJ, Van Oortmarssen GJ, Vos T, et al. A generic model for the assessment of disease epidemiology: the computational basis of DisMod II. *Popul Health Metr* 2003;1(1):4.

33. Peng R. The reproducibility crisis in science: A statistical counterattack. *Significance* 2015;**12**(3):30-32.

34. Gunning-Schepers L. The health benefits of prevention: a simulation approach. *Health Policy* 1989;**12**(1-2):1-255.

35. Moher D, Liberati A, Tetzlaff J, et al. Preferred reporting items for systematic reviews and meta-analyses: the PRISMA statement. *PLoS Med* 2009;6(7):e1000097.

For peer review only - http://bmjopen.bmj.com/site/about/guidelines.xhtml

For peer review only

36. Stevens GA, Alkema L, Black RE, et al. Guidelines for Accurate and Transparent Health Estimates Reporting: the GATHER statement. *Lancet (London, England)* 2016;388(10062):e19-e23.

37. Philips Z, Bojke L, Sculpher M, et al. Good practice guidelines for decision-analytic modelling in health technology assessment: a review and consolidation of quality assessment. *Pharmacoeconomics* 2006;24(4):355-71.

38. Weinstein MC, Toy EL, Sandberg EA, et al. Modeling for health care and other policy decisions: uses, roles, and validity. *Value Health* 2001;4(5):348-61.

39. Shamseer L, Moher D, Clarke M, et al. Preferred reporting items for systematic review and meta-analysis protocols (PRISMA-P) 2015: elaboration and explanation. *BMJ* 2015;349:g7647.

For peer review only

For peer review only - http://bmjopen.bmj.com/site/about/guidelines.xhtml

Supplementary APPENDIX

Search Strategies Developed for MEDLINE

1. exp Asthma/
2. Respiratory Sounds/
3. wheez$.mp.
4. Bronchial Spasm/
5. bronchospas$.mp.
6. (bronch$ adj3 spasm$).mp.
7. bronchoconstrict$.mp.
8. asthma$.mp.
9. (antiasthma$ or anti-asthma$).mp.
10. exp Bronchoconstriction/
11. (bronch$ adj3 constrict$).mp.
12. Bronchial Hyperreactivity/
13. Respiratory Hypersensitivity/
14. ((bronchial$ or respiratory or airway$ or lung$) adj3 (hypersensitiv$ or hyperreactiv$ or allerg$ or insufficiency)).mp.
15. or/1-14
16. Epidemiology/
17. morbidity/ or incidence/ or prevalence/ or mortality/
18. (disability-adjusted life years or disability adjusted life years or DALY$).mp.
19. Quality-Adjusted Life Years/ or (quality adjusted life years or QALY$).mp.
20. years lived with disability.mp.
21. years life lost.mp.
22. potential years of life lost.mp.

For peer review only - http://bmjopen.bmj.com/site/about/guidelines.xhtml

23. healthy years of life lost.mp.
24. active life expectancy.mp.
25. (disability-free life expectancy or disability free life expectancy).mp.
26. (disability-adjusted life expectancy or disability adjusted life expectancy).mp.
27. (Healthy life expectancy or HALE).mp.
28. (quality adjusted life expectancy or quality-adjusted life expectancy).mp.
29. vital statistics/ or life expectancy/ or life tables/
30. "costs and cost analysis"/ or "cost of illness"/ or health care costs/ or drug costs/ or hospital costs/ or health expenditures/
31. Hospitalization/ or hospitalisation.mp.
32. Primary Health Care/ or Ambulatory Care/ or Emergency Service, Hospital/ or absentee$.mp.
33. burden$.mp.
34. or/16-33
35. model$.mp. or exp models, statistical/ or exp regression analysis/ or odds ratio/ or monte carlo method/ or markov chains/ or Markov.mp.
36. (estimat$ or projection$ or projecting).ti.
37. exp Forecasting/
38. or/35-37
39. 15 and 34 and 38

For peer review only

For peer review only - http://bmjopen.bmj.com/site/about/guidelines.xhtml

Search Strategies Developed for EMBASE

1. exp asthma/
2. asthma$.mp.
3. (antiasthma$ or anti-asthma$).mp.
4. abnormal respiratory sound/
5. wheez$.mp.
6. bronchospasm/
7. bronchospas$.mp.
8. (bronch$ adj3 spasm$).mp.
9. bronchoconstrict$.mp.
10. bronchoconstriction/
11. (bronch$ adj3 constrict$).mp.
12. bronchus hyperreactivity/
13. respiratory tract allergy/
14. ((bronchial$ or respiratory or airway$ or lung$) adj3 (hypersensitiv$ or hyperreactiv$ or allerg$ or insufficiency)).mp.
15. or/1-14
16. epidemiology/
17. Prevalence/ or Incidence/ or Morbidity/ or Mortality/
18. burden$.mp.
19. (disability-adjusted life years or disability adjusted life years or DALY$).mp.
20. quality adjusted life year/ or (quality-djusted life years or QALY$).mp.
21. years lived with disability.mp.
22. years life lost.mp.
23. potential years of life lost.mp.

For peer review only

For peer review only - http://bmjopen.bmj.com/site/about/guidelines.xhtml

24. healthy years of life lost.mp.

25. Vital statistics/ or Life expectancy/ or Life tables/

26. active life expectancy.mp.

27. (disability-free life expectancy or disability free life expectancy).mp.

28. (disability-adjusted life expectancy or disability adjusted life expectancy).mp.

29. (Healthy life expectancy or HALE).mp.

30. (quality adjusted life expectancy or quality-adjusted life expectancy).mp.

31. "health care cost"/ or "drug cost"/ or "hospital cost"/ or "hospitalization cost"/ or "cost"/ or "cost of illness"/

32. hospitalization/ or Hospitalisation.mp.

33. primary health care/ or medical care/ or emergency care/ or emergency treatment/ or emergency health service/ or ambulatory care/ or absenteeism/

34. or/16-33

35. model/ or loglinear model/ or population model/ or mathematical model/ or proportional hazards model/ or statistical model/ or stochastic model/ or markov chain/ or Markov.mp.

36. (estimat$ or projection$ or projecting).mp.

37. "prediction and forecasting"/

38. or/35-37

39. 15 and 34 and 38

For peer review only

Model evaluation checklist and scoring framework

Checklist

Checklist item	Score obtained (Yes = 1, No = 0)
**Strength**	
**Objective/purpose:**<br>1. Whether there is clear statement about the questions that the model aimed to answer	
**Model formulation and transparency:**<br>Whether there is clear description about<br>1. model structure or model statement (including formula)<br>2. model specification<br>3. model assumptions<br>4. model derivation<br>5. variable used in the model (response, predictors, potential confounders, lag time)<br>6. method of parameter estimation and inference<br>7. model building (variable selection) process<br>8. model diagnostic and adequacy checking<br>9. theoretical ground of the model	
**Data:**<br>Whether there is clear description about<br>1. source of data<br>2. method of data collection (study design)<br>3. sample size and determination process<br>4. method of assessment (data measurement)	
**Model findings:**<br>Whether following information are available<br>1. necessary estimates (prevalence/burden measure/incidence)<br>2. standard errors and 95% confidence intervals of the estimates<br>3. model fit information<br>4. compliance of the model findings with real scenario	
**Model validation:**<br>1. Whether model validation was performed	
**Limitation**	
**Sensitivity analysis:**<br>1. Whether sensitivity analyses was carried out	
**Addressing missing data**<br>1. Whether there is explanation about how missing data were addressed	
**Dissemination and expert involvement:**<br>1. Whether model was disseminated prior to final development of model<br>2. Whether expert opinion were incorporated to develop the model	
**Policymakers involvement:**<br>1. Whether policymakers were involved in the model development<br>2. Whether any policy was recommended on the basis of model derived findings	
**Limitation discussed in the paper:**<br>Whether potential methodological limitations of the model were discussed	

For peer review only

Reproducibility	
**Reproducibility:**<br>1. Whether model is available and accessible to user<br>2. Whether data are available<br>3. Whether codes are available<br>4. Whether user manual to develop the model is available	

Scoring framework

Criteria	Item	Score assigned	Score obtained
Strength	Objectives of the model	1	
	Model formulation and transparency	9	
	Data	4	
	Model findings	4	
	Model validation	1	
Limitation	Sensitivity analysis	1	
	Addressing missing data	1	
	Dissemination and expert involvement	2	
	Policymakers involvement	2	
	Limitation discussed in the paper	1	
Reproducibility	Reproducibility of the model	4	
Total		30	

For peer review only

PRISMA-P (Preferred Reporting Items for Systematic review and Meta-Analysis Protocols) 2015 checklist: recommended items to address in a systematic review protocol*

Section and topic	Item No	Checklist item	
**ADMINISTRATIVE INFORMATION**			
Title:			
Identification	1a	Identify the report as a protocol of a systematic review	1
Update	1b	If the protocol is for an update of a previous systematic review, identify as such	NA
Registration	2	If registered, provide the name of the registry (such as PROSPERO) and registration number	NA
Authors:			
Contact	3a	Provide name, institutional affiliation, e-mail address of all protocol authors; provide physical mailing address of corresponding author	1
Contributions	3b	Describe contributions of protocol authors and identify the guarantor of the review	12
Amendments	4	If the protocol represents an amendment of a previously completed or published protocol, identify as such and list changes; otherwise, state plan for documenting important protocol amendments	NA
Support:			
Sources	5a	Indicate sources of financial or other support for the review	12
Sponsor	5b	Provide name for the review funder and/or sponsor	12
Role of sponsor or funder	5c	Describe roles of funder(s), sponsor(s), and/or institution(s), if any, in developing the protocol	NA
**INTRODUCTION**			
Rationale	6	Describe the rationale for the review in the context of what is already known	4-5
Objectives	7	Provide an explicit statement of the question(s) the review will address with reference to participants, interventions, comparators, and outcomes (PICO)	6
**METHODS**			
Eligibility criteria	8	Specify the study characteristics (such as PICO, study design, setting, time frame) and report characteristics (such as years considered, language, publication status) to be used as criteria for eligibility for the review	6-7
Information sources	9	Describe all intended information sources (such as electronic databases, contact with study authors, trial registers or other grey literature sources) with planned dates of coverage	7
Search strategy	10	Present draft of search strategy to be used for at least one electronic database, including planned limits, such that it could be repeated	8, 18-21
Study records:			
Data management	11a	Describe the mechanism(s) that will be used to manage records and data throughout the review	8

For peer review only

Selection process	11b	State the process that will be used for selecting studies (such as two independent reviewers) through each phase of the review (that is, screening, eligibility and inclusion in meta-analysis)	8
Data collection process	11c	Describe planned method of extracting data from reports (such as piloting forms, done independently, in duplicate), any processes for obtaining and confirming data from investigators	8
Data items	12	List and define all variables for which data will be sought (such as PICO items, funding sources), any pre-planned data assumptions and simplifications	9
Outcomes and prioritization	13	List and define all outcomes for which data will be sought, including prioritization of main and additional outcomes, with rationale	9-10
Risk of bias in individual studies	14	Describe anticipated methods for assessing risk of bias of individual studies, including whether this will be done at the outcome or study level, or both; state how this information will be used in data synthesis	10-11
Data synthesis	15a	Describe criteria under which study data will be quantitatively synthesised	11
	15b	If data are appropriate for quantitative synthesis, describe planned summary measures, methods of handling data and methods of combining data from studies, including any planned exploration of consistency (such as I^2, Kendall's τ)	11
	15c	Describe any proposed additional analyses (such as sensitivity or subgroup analyses, meta-regression)	NA
	15d	If quantitative synthesis is not appropriate, describe the type of summary planned	11
Meta-bias(es)	16	Specify any planned assessment of meta-bias(es) (such as publication bias across studies, selective reporting within studies) NA	
Confidence in cumulative evidence	17	Describe how the strength of the body of evidence will be assessed (such as GRADE)	NA

*** It is strongly recommended that this checklist be read in conjunction with the PRISMA-P Explanation and Elaboration (cite when available) for important clarification on the items. Amendments to a review protocol should be tracked and dated. The copyright for PRISMA-P (including checklist) is held by the PRISMA-P Group and is distributed under a Creative Commons Attribution Licence 4.0.**

From: Shamseer L, Moher D, Clarke M, Ghersi D, Liberati A, Petticrew M, Shekelle P, Stewart L, PRISMA-P Group. Preferred reporting items for systematic review and meta-analysis protocols (PRISMA-P) 2015: elaboration and explanation. BMJ. 2015 Jan 2;349(jan02 1):g7647.

For peer review only
